# Can a mosquito-borne virus become a cancer therapy? rethinking Getah virus through the lens of oncolytic virotherapy

**DOI:** 10.3389/fcimb.2026.1768174

**Published:** 2026-06-03

**Authors:** Bernard B. Efa, Jacob Antwi-Osei Jnr

**Affiliations:** 1 Microbiology Program, Department of Biological Sciences, College of Science, Technology, Engineering and Mathematics, Alabama State University, Montgomery, AL, United States; 2 Master of Public Health (MPH) Program, University of Delaware, Newark, DE, United States

**Keywords:** alphavirus, Getah virus, M1, MM2021, mosquito, oncolytic virus

## Abstract

Getah virus (GetV) is an arthropod-borne alphavirus historically recognized as an emerging zoonotic pathogen of veterinary significance, particularly in livestock and equine populations across Asia and parts of the Western Pacific. Over the past several decades, its expanding ecological range, broad mosquito vector competence, and increasing frequency of animal outbreaks have positioned GetV as a growing concern for animal health surveillance, diagnostics, and vaccine development. However, beyond its established role in veterinary virology, a critical and underexplored dimension of GetV biology is its emerging potential in oncolytic virotherapy. Recent discoveries, particularly involving the M1 strain, reveal a striking capacity for tumor-selective replication driven by defects in antiviral innate immune signaling within malignant cells. This property positions GetV-derived platforms as promising candidates for next-generation oncolytic virus development, capable of direct tumor lysis and secondary activation of antitumor immunity. These findings signal a paradigm shift in how traditionally zoonotic alphaviruses may be repurposed for precision oncology. We therefore hypothesize that whilst broad cellular tropism enables Getah virus entry into different kinds of cells, the oncolytic efficacy requires another layer of intracellular permissiveness characterized by tumor-specific innate immune defects. This Perspective synthesizes the current state of knowledge on GetV from both veterinary and translational oncology viewpoints and outlines the dual-use trajectory of the virus from agricultural pathogen to therapeutic bioplatform. We further highlight unresolved questions surrounding mechanisms of tumor selectivity, biosafety and host restriction, genetic stability, immune modulation, and regulatory translational barriers. Addressing these gaps will be essential for advancing GetV-based oncolytic platforms toward clinical applicability. Collectively, GetV represents a compelling example of how emerging zoonotic viruses may be strategically repositioned at the interface of infectious disease surveillance and cancer therapy innovation.

## Introduction

Getah virus (GetV) was first isolated in Malaysia in 1955 and is primarily transmitted through a mosquito–vertebrate–mosquito transmission cycle typical of alphaviruses (Li et al., 2022). It is found in nature as a single stranded, positive-sense RNA virus. It belongs to the genus Alphavirus of the family Togaviridae with Chikungunya virus, Sindbis virus, and Ross River virus as members of this family (Lin et al., 2014; Guo et al., 2022). GETV has two open reading frames that code for both structural and non-structural proteins (nsp1–nsp4). The E2 glycoprotein is an important feature of how the virus enters into host cells (Lin et al., 2014; Guo et al., 2022). Over time, GetV has grown a lot in both the places it can infect and the types of hosts it can infect. It has been found in horses, pigs, cows, wild boars, foxes, and numerous types of mosquitoes, such as Culex, Aedes, Anopheles, and Mansonia (Huang et al., 2019; Shi et al., 2022; Shen et al., 2025; Deng et al., 2025; Jittprasong, 2022). Serological surveillance shows that animals are exposed to the disease a lot, and that blood antibodies rise a lot during epidemics. Serological studies of the Getah virus (GETV) show a global average seroprevalence of 33.3% in animals. GETV seroprevalence varies widely across studies, ranging from moderate to high levels in endemic animal populations (Sun et al., 2022; Huang et al., 2025; Irekeola and Shueb, 2025). Current data suggests that exposure, especially in livestock, increases significantly during peak mosquito seasons, often exceeding 50% in affected regions (Sun et al., 2022; Huang et al., 2025; Irekeola and Shueb, 2025). Seropositivity in humans has been recorded in many regions of Asia, notably China and Malaysia; nevertheless, a definitive molecular diagnosis of active human infection remains unconfirmed. Also, during and after Getah virus outbreaks, animal populations show a dramatic shift toward high-titer brackets, with 60.9% of positive beef cattle in one study and 20.5% of positive pigs in a Yunnan study exceeding 1:640 levels (Sun et al., 2022; Takeishi et al., 2022; Irekeola and Shueb, 2025; Lan et al., 2024). These high antibody levels (specifically) are remarkably persistent, as seen in Japanese horses study where protection lasted at least three years without reinfection (Sun et al., 2022; Takeishi et al., 2022; Irekeola and Shueb, 2025). Phylogenetic analyses of the E2 gene categorize GetV into four primary groups, with Group III identified as the principal and epidemiologically relevant branch (Li et al., 2022; Tamura et al., 2021; Yuan et al., 2017; Zhao et al., 2023).

Although mosquito transmission is dominant, aerosol transmission has been proposed, underscoring potential public health implications (Shen et al., 2025; Jian et al., 2024; Jittprasong, 2022). Vaccination strategies have relied primarily on inactivated formulations. While formalin-inactivated vaccines show efficacy against classical strains, oil-emulsion inactivated vaccines appear to confer broader and more robust protection in experimental models (Swine Health Information Center, 2021). Nevertheless, the continued emergence of novel strains raises concerns regarding long-term vaccine effectiveness (Li et al., 2017; Shen et al., 2025).

**Figure 1:**
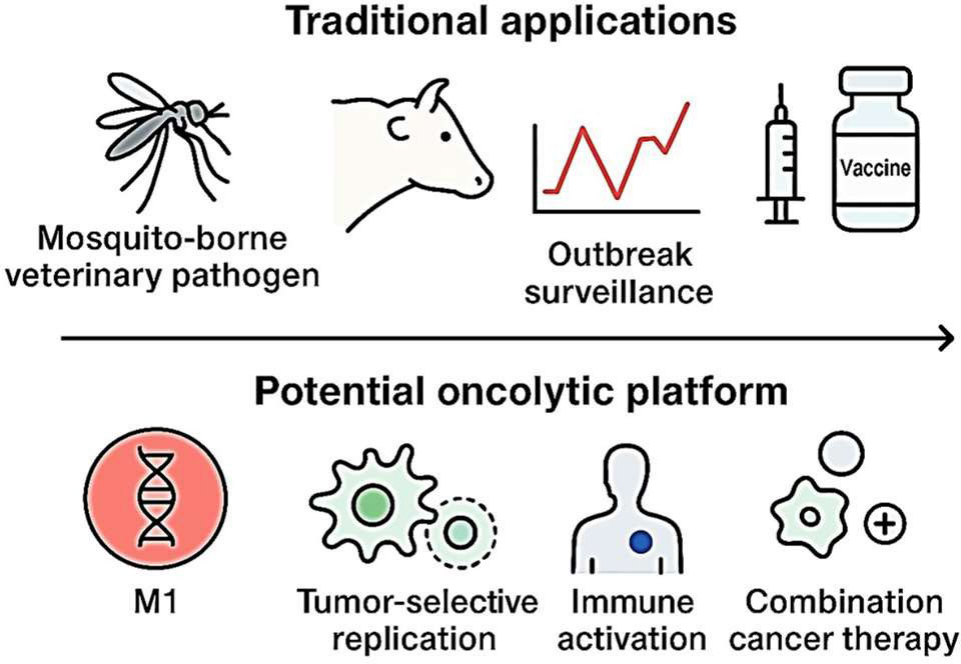
A diagram showing how the perception of the Getah virus has changed from being a mosquito-borne veterinary pathogen linked to cattle infection, outbreak surveillance, and vaccine development to its new status as a possible oncolytic platform represented via the M1 strain.

Against this expanding backdrop of zoonotic and veterinary significance, the biological features that enable GetV to efficiently enter, replicate, and spread in diverse hosts also provide a mechanistic bridge to its emerging role in cancer therapy (**Figure 1**). Specifically, traits traditionally viewed as liabilities for animal health—robust replication, broad cellular tropism, and rapid cytolysis—are the very characteristics sought in effective oncolytic viruses (Zhang et al., 2017; Chen and Guo, 2023; Sułek and Szuster-Ciesielska, 2025). It is within this translational framework that the identification of the M1 strain as a selective oncolytic virus represents a pivotal conceptual shift (Lin et al., 2014; Meng et al., 2014; Zhang et al., 2017; Lin et al., 2023). M1 exhibits preferential replication in malignant cells with defective antiviral signaling (e.g., ZAP deficiency), leading to efficient tumor cell lysis while largely sparing normal tissues (Lin et al., 2014; Meng et al., 2014; Zhang et al., 2017; Lin et al., 2023). Importantly, this selective cytopathicity not only mediates direct tumor debulking but also promotes secondary antitumor immunity through the release of tumor antigens and danger-associated molecular patterns within the tumor microenvironment (Cai et al., 2020; Cai et al., 2021). Thus, GetV-related strains are no longer viewed solely as pathogens but as programmable biological agents with dual cytolytic and immunostimulatory capacity (Chen and Guo, 2023; Sułek and Szuster-Ciesielska, 2025).

Nevertheless, the therapeutic promise of M1 simultaneously exposes a fundamental limitation in the current knowledge base: it remains unresolved whether oncolytic activity is an exceptional property of this single strain or a latent feature distributed across the broader genetic landscape of GetV (Lin et al., 2014; Meng et al., 2014; Feola et al., 2023; Jenner et al., 2019). Resolving this question is essential for rational platform development. Progress in this direction will require systematic dissection of (i) the viral genetic determinants that govern tumor selectivity and host-range restriction (Guo et al., 2022; Wang et al., 2022a, b), (ii) standardized *in vitro* and *in vivo* models that enable direct comparison of candidate GetV strains under uniform experimental conditions (Hemminki et al., 2020; Qi et al., 2024; Ramaj and Zou, 2023; Wang et al., 2022a, b; Zeng et al., 2021), (iii) long-term biosafety profiling to exclude delayed cytopathicity, neurotropism, or persistence in normal tissues (Hemminki et al., 2020; Chen and Guo, 2023) and (iv) the biological consequences of GetV-based oncolysis in hosts bearing multiple, genetically heterogeneous malignancies (Chen and Guo, 2023; Sułek and Szuster-Ciesielska, 2025).

Equally important is the temporal dimension of oncolytic evaluation (Mondal et al., 2020). Current *in vitro* studies frequently emphasize short-term infection windows (24–96 h), which may adequately capture acute cytolytic effects but are insufficient to model delayed toxicity, viral persistence, or durable antitumor immunity (Cai et al., 2021; Chen and Guo, 2023). Extending infection kinetics to later time points will be essential for defining therapeutic index, safety margins, and true translational feasibility. Together, these transition points delineate the pathway by which GetV may evolve from an emerging zoonotic virus into a rigorously validated oncolytic platform (Lin et al., 2014; Meng et al., 2014; Zhang et al., 2017).

**Figure 2:**
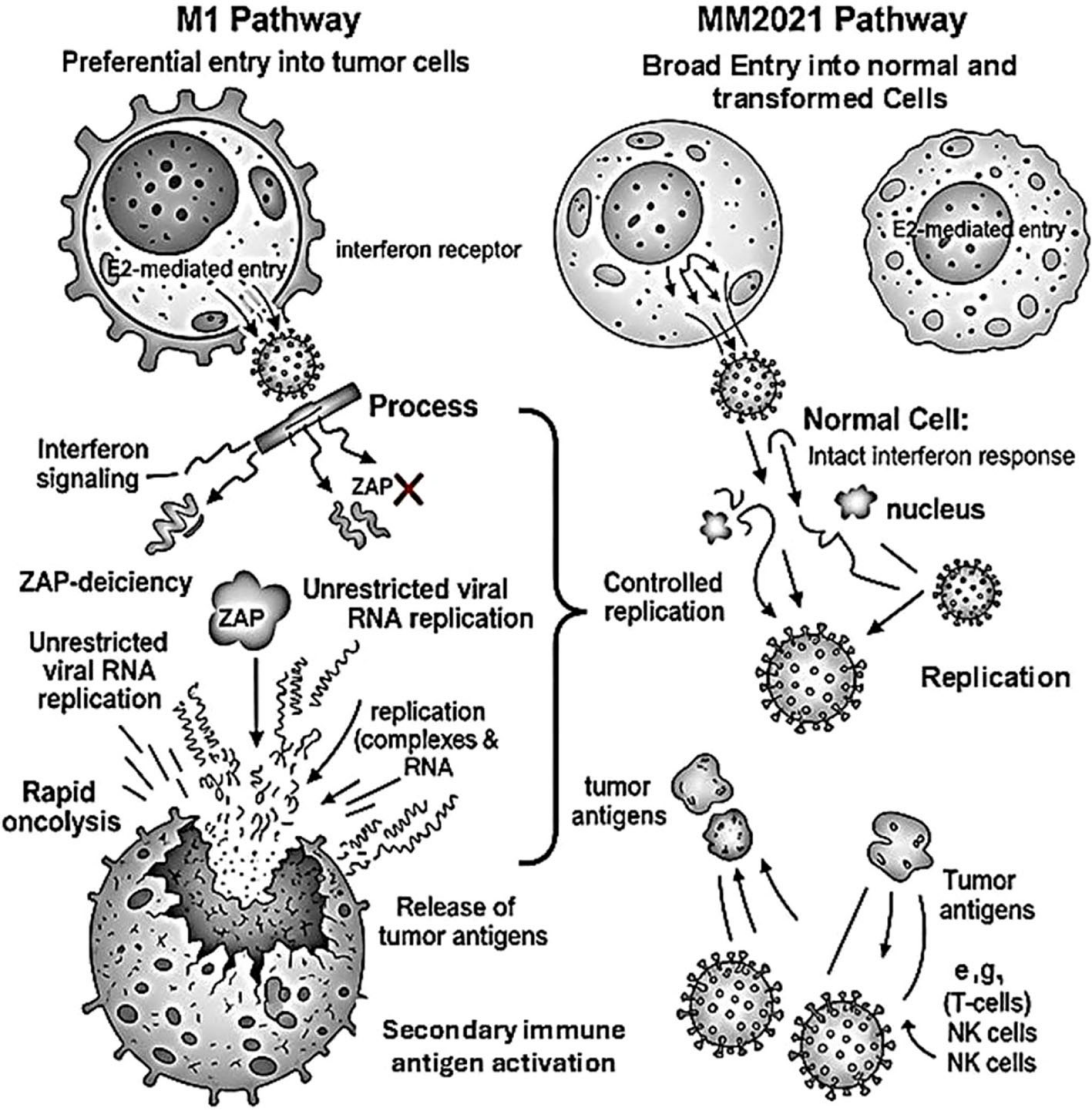
Mechanistic comparison of M1 and MM2021 Getah Virus strains. The M1 strain preferentially infects tumor cells with defective interferon signaling and ZAP deficiency, enabling unrestricted viral RNA replication, rapid oncolysis, and tumor antigen release. In contrast, the MM2021 strain exhibits broad cellular tropism, infecting both normal and transformed cells with preserved interferon responses, resulting in controlled replication and productive, non-selective infection.

**Figure 3:**
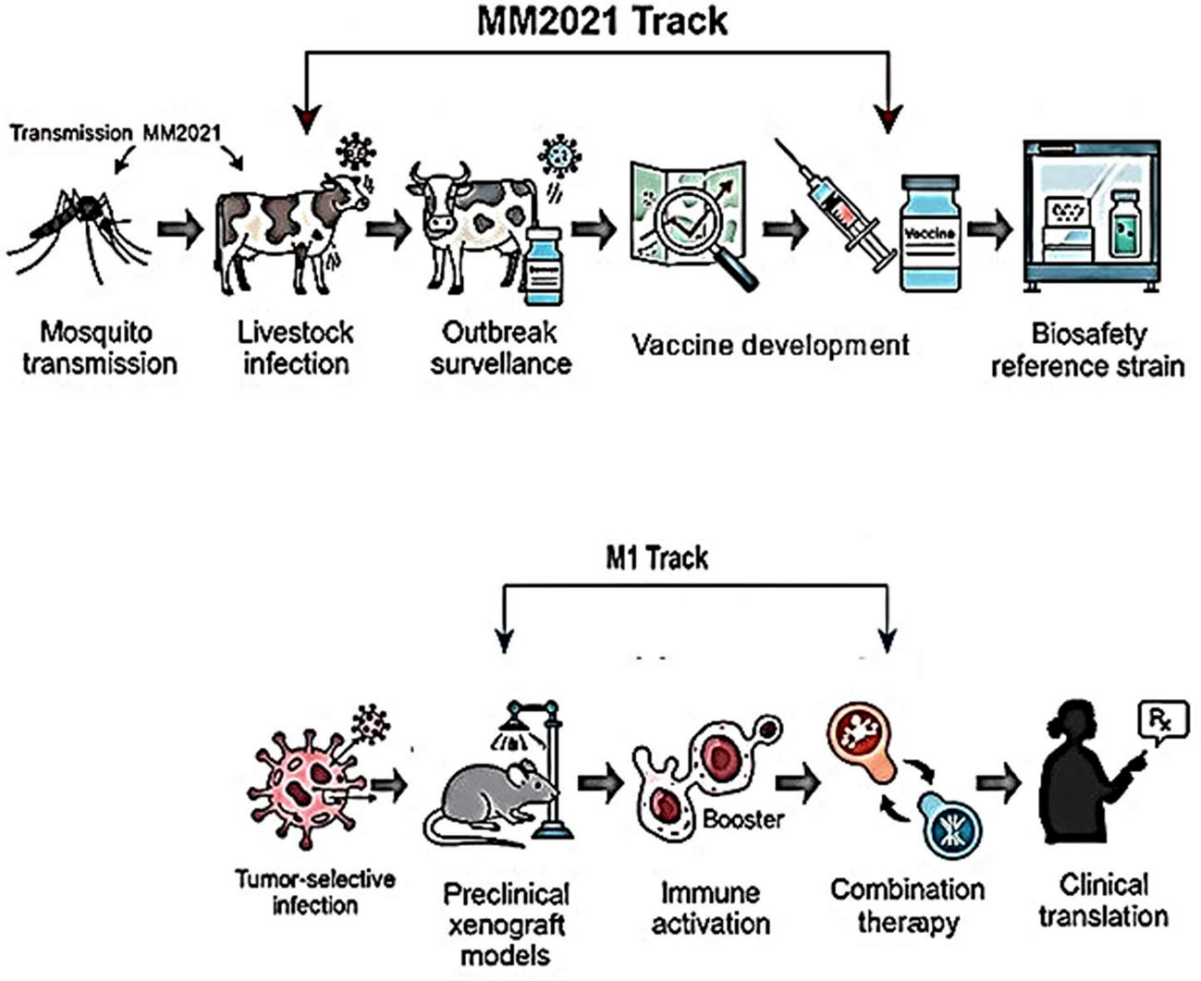
Translational application pipeline: veterinary pathogen to oncolytic platform. The translational framework of Getah virus highlights a dual-use pipeline separating veterinary outbreak control from oncolytic development. The veterinary trajectory, represented by the MM2021 strain, follows mosquito-driven transmission, livestock infection, molecular surveillance, and progression toward vaccine development and biosafety reference standardization. In contrast, the oncolytic trajectory, defined by the M1 strain, is characterized by tumor-selective replication, validation in xenograft models, induction of antitumor immunity, and evaluation for combination therapy and clinical translation. Together, these pathways emphasize a shift from broad host-range pathogenicity to tumor-restricted targeting under increasingly stringent safety and regulatory barriers.

Building on this framework, the MM2021 GetV strain warrants special mechanistic consideration as a comparative biosafety and host-range reference for oncolytic development. MM2021 is phylogenetically aligned with contemporary epidemic GetV lineages and displays efficient replication in mosquito and mammalian cells, indicating intact entry, replication, and egress machinery (Lin et al., 2014; Guo et al., 2022; Li et al., 2022). At the cellular level, its life cycle is governed by classical alphaviral processes: E2-mediated receptor attachment and endocytosis, pH-dependent membrane fusion within endosomes, rapid RNA replication driven by the nsp1–nsp4 complex, and structural polyprotein processing for virion assembly and budding (Lin et al., 2014; Guo et al., 2022). Unlike M1, however, MM2021 has not been shown to exhibit intrinsic selectivity for antiviral-defective tumor cells, suggesting that broad replicative competence alone is insufficient to confer oncolytic specificity (Lin et al., 2014; Meng et al., 2014; Zhang et al., 2017). This distinction is mechanistically important because it implies that tumor selectivity in M1 likely arises from discrete genomic features affecting innate immune antagonism, interferon sensitivity, or intracellular RNA restriction pathways rather than from generic alphavirus tropism (Lin et al., 2014; Meng et al., 2014; Zhang et al., 2017). Consequently, MM2021 provides a valuable experimental baseline for dissecting the precise genetic determinants that must be engineered or selected to convert a naturally pathogenic GetV strain into a safe, tumor-restricted oncolytic vector (Lin et al., 2014; Meng et al., 2014; Zhang et al., 2017). These have been summarized in the **Figures 2** and **3** with further comparisons between M1 and MM2021 depicted in **Table 1**

**Table 1:** Comparative features of M1 and MM2021 Getah Virus strains.

Feature	M1 strain	MM2021 strain
Primary Biological Role	Oncolytic candidate	Epidemic zoonotic strain
Cell Tropism	Preferential for tumor cells	Broad mammalian + mosquito cells
Interferon Sensitivity	Reduced	Preserved
ZAP-Deficiency Dependence	Yes	Not demonstrated
Tumor Selectivity	High	None demonstrated
Clinical Application	Experimental cancer therapy	Veterinary pathogen, vaccine target
Biosafety Role	Therapeutic test vector	Reference pathogenic strain

## Conclusion

Getah virus is firmly established as a zoonotic alphavirus of growing veterinary significance; however, the emergence of the M1 strain as a selective oncolytic virus fundamentally broadens its biomedical relevance. This transition from pathogen to potential therapeutic underscores the importance of reframing future GetV research within a translational oncology context. Focused investigations into strain specificity, biosafety, host immunity, and standardized therapeutic evaluation are essential before broader clinical translation is feasible. If these challenges are systematically addressed, additional GetV strains may ultimately be positioned as novel candidates within the next generation of targeted cancer virotherapies.

## Data Availability

The original contributions presented in the study are included in the article/supplementary material. Further inquiries can be directed to the corresponding author.
